# GABA_a_ excitation and synaptogenesis after *Status Epilepticus* – A computational study

**DOI:** 10.1038/s41598-018-22581-6

**Published:** 2018-03-08

**Authors:** Keite Lira de Almeida França, Antônio-Carlos Guimarães de Almeida, Stephen E. Saddow, Luiz Eduardo Canton Santos, Carla Alessandra Scorza, Fulvio Alexandre Scorza, Antônio Márcio Rodrigues

**Affiliations:** 1 Laboratório de Neurociência Experimental e Computacional (LANEC), Departamento de Engenharia de Biossistemas, Universidade Federal de São João del-Rei (UFSJ), São João del-Rei, Brazil; 2 Electrical Engineering Department, University of South of Florida, Tampa, FL USA; 3 Disciplina de Neurologia Experimental, Escola Paulista de Medicina, Unifesp, Brazil

## Abstract

The role of GABAergic neurotransmission on epileptogenesis has been the subject of speculation according to different approaches. However, it is a very complex task to specifically consider the action of the GABAa neurotransmitter, which, in its dependence on the intracellular level of Cl^−^, can change its effect from inhibitory to excitatory. We have developed a computational model that represents the dentate gyrus and is composed of three different populations of neurons (granule cells, interneurons and mossy cells) that are mutually interconnected. The interconnections of the neurons were based on compensation theory with Hebbian and anti-Hebbian rules. The model also incorporates non-synaptic mechanisms to control the ionic homeostasis and was able to reproduce ictal discharges. The goal of the work was to investigate the hypothesis that the observed aberrant sprouting is promoted by GABAa excitatory action. Conjointly with the abnormal sprouting of the mossy fibres, the simulations show a reduction of the mossy cells connections in the network and an increased inhibition of the interneurons as a response of the neuronal network to control the activity. This finding contributes to increasing the changes in the connectivity of the neuronal circuitry and to increasing the epileptiform activity occurrences.

## Introduction

Epilepsy is the most common chronic neurological disorder, and it is characterized by hyper-synchronization and hyper-excitability of the neuronal network^1,2^. The hyper-excitability is normally interpreted as an under-regulation of the synaptic inhibition^3^, which promotes unbalancing between excitation and inhibition. However, recent research shows that excitation/inhibition balance is not the solely cause of the development and aetiology of epilepsies^4^.

In fact, it has been shown that the hyper-synchronism observed in epilepsy is associated with an enhancement of the mutual neuronal coupling^4–6^. According to the most widely accepted theory, abnormal mossy fibre sprouting (AMFS) promotes mutual coupling of the granule cells of the hippocampal dentate gyrus, which in turn is responsible for hyper-excitability of these cells^7–10^.

On the other hand, in the centre of the discussion about the unbalancing between excitation and inhibition is GABAergic neurotransmission. It is known that the GABAa neurotransmission can act by either inhibition or excitation, depending on the intracellular Cl^−^ concentration [Cl^−^]_i_^4^. Low [Cl^−^]_i_ results in a negative shift of the GABAa reverse potential (E_GABAa_) with respect to the membrane potential, which affords an inhibitory action and serves as a dampening function in the brain. High [Cl^−^]_i_ promotes a positive shift in the E_GABAa_, which exhibits an excitatory effect on the post-synaptic membrane. In hippocampal neurons, the [Cl^−^]_i_ level is dictated by the two major Cl^−^ regulators: the Na^+^-K^+^-2Cl^−^ cotransporter (NKCC) and the K^+^Cl^−^ cotransporter (KCC). Decreased expression of KCC has been shown in granule cells after an epileptogenic injury, which is a condition that has a significant effect in favour of intracellular Cl^−^ accumulation and in the subsequent development of epilepsy^11^.

*Status epilepticus* (SE) is a brain insult that is characterized by a prolonged seizure or multiple seizures with incomplete return to baseline^8^. Experimentally, it is induced with a chemical or electrical stimulus application with enough intensity to produce excessive and abnormal excitation and a failure of the mechanisms that can terminate a seizure. The interval between the initial brain insult and the manifestation of the first spontaneous seizure is called the latent period, and the duration of the period depends on the protocol used in the experimental model and can last for days to months. Investigations show that in the course of that latent period, several pathophysiological phenomena that lead to epileptogenesis can occur^8,9^, like changing in the expression of cotransporters associated with the inflammatory process, reflecting accumulation of intracellular chloride, and also cellular swelling, leading to enhanced non-synaptic interconnections. This pathophysiological scenario is particularly favourable to the excitation/inhibition unbalancing and mutual neuronal coupling.

In order to investigate how abnormal mossy fibre sprouting can subserve seizure generation during the latent period, recently, we introduced a computational model that represents the dentate gyrus (DG) composed of three populations of neurons (granule cells, interneurons and mossy cells) that are mutually interconnected^10^. The synaptogenesis process that governs changes in the neuronal cell interconnections is dependent on the neuronal activity and was based on compensation theory and the Hebbian and anti-Hebbian rules. The model also incorporates non-synaptic mechanisms that are related to changes in the ionic homeostasis, which allows reproducing epileptiform discharge. The first version of the model was useful to test the feasibility of the proposed formalism to represent synaptogenesis in the circumstances of the neuronal network changes during epileptiform activity.

In the present work, we investigated the effect of the changes in the duration of the period in which the GABAa remains excitatory and also in the number of granular cells that underwent inversion of the GABA_a_ effect (from inhibitory to excitatory). Comparing the simulations with experimental observations reported in the literature^11–14^ the study allows proposing the hypothesis that excitation/inhibition unbalancing is linked to increased neuronal mutual coupling, more specifically, that the observed aberrant sprouting is promoted by the GABAa excitatory effect.

## Results

**Figure 1:**
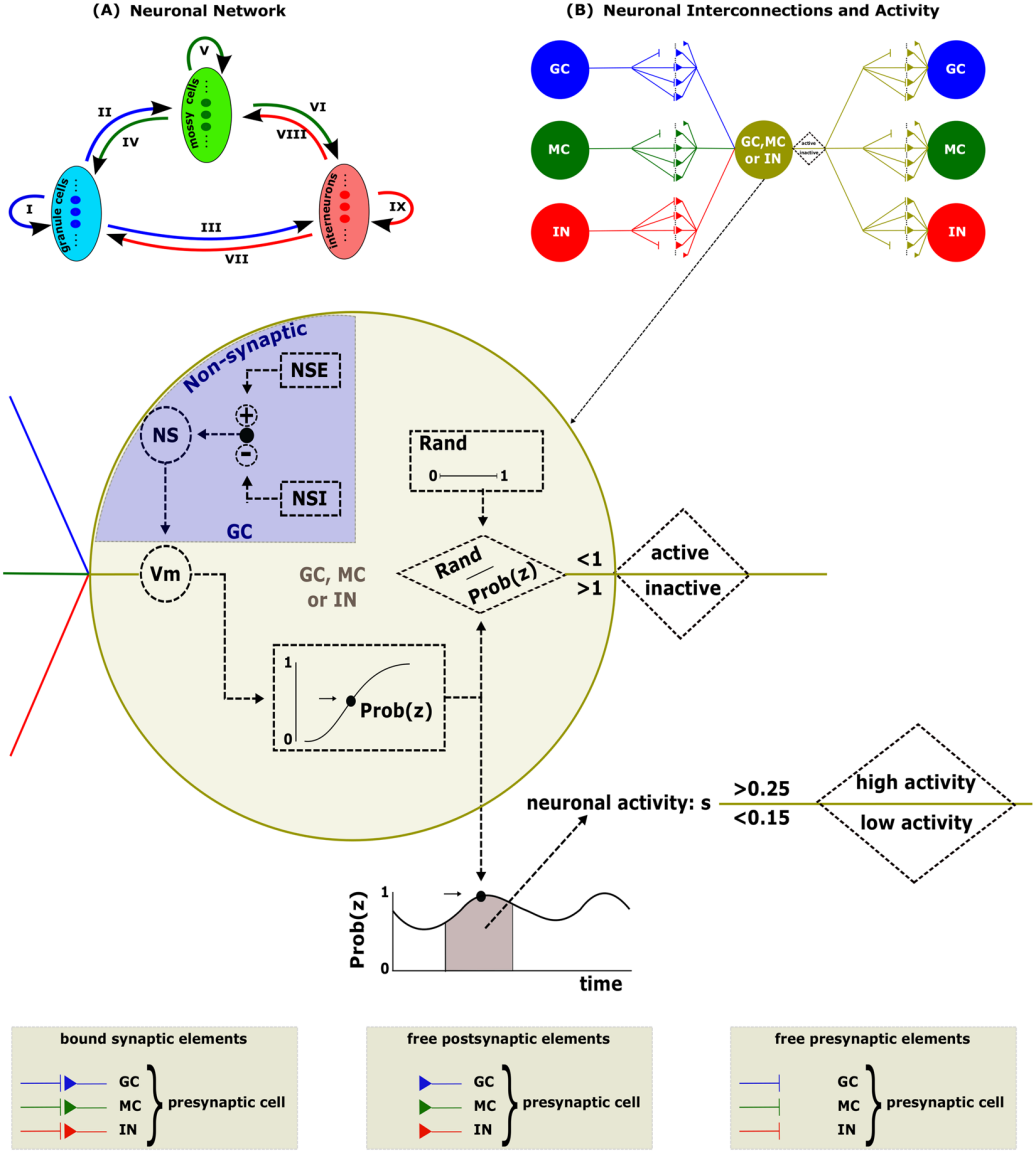
(**A**) Schematic representation of the neural network model. Granule cell (GC), mossy cell (MC) and interneuron (IN). The granule and mossy cells provide excitatory input to the other cells, whereas the interneurons are inhibitory. The three cell types form 9 connectivity groups: I, II, III, IV, V, VI, VII, VIII and IX. (**B**) Neural connections and calculation of cellular activity. A neuron receives impulses from other neurons in the network, thus increasing its probability of firing Prob(z), which is dependent on the membrane potential (Vm). For GC and MC, the effect of non-synaptic mechanisms (NS), excitatory (NSE) and inhibitory (NSI) components on the membrane potential were considered. Changes in the mean neuronal activity (hatching area of the curve) induce changes in the functional status (low or high state of activity). The changes in the activity lead to the formation or decay of different types of synaptic elements.

**Figure 2:**
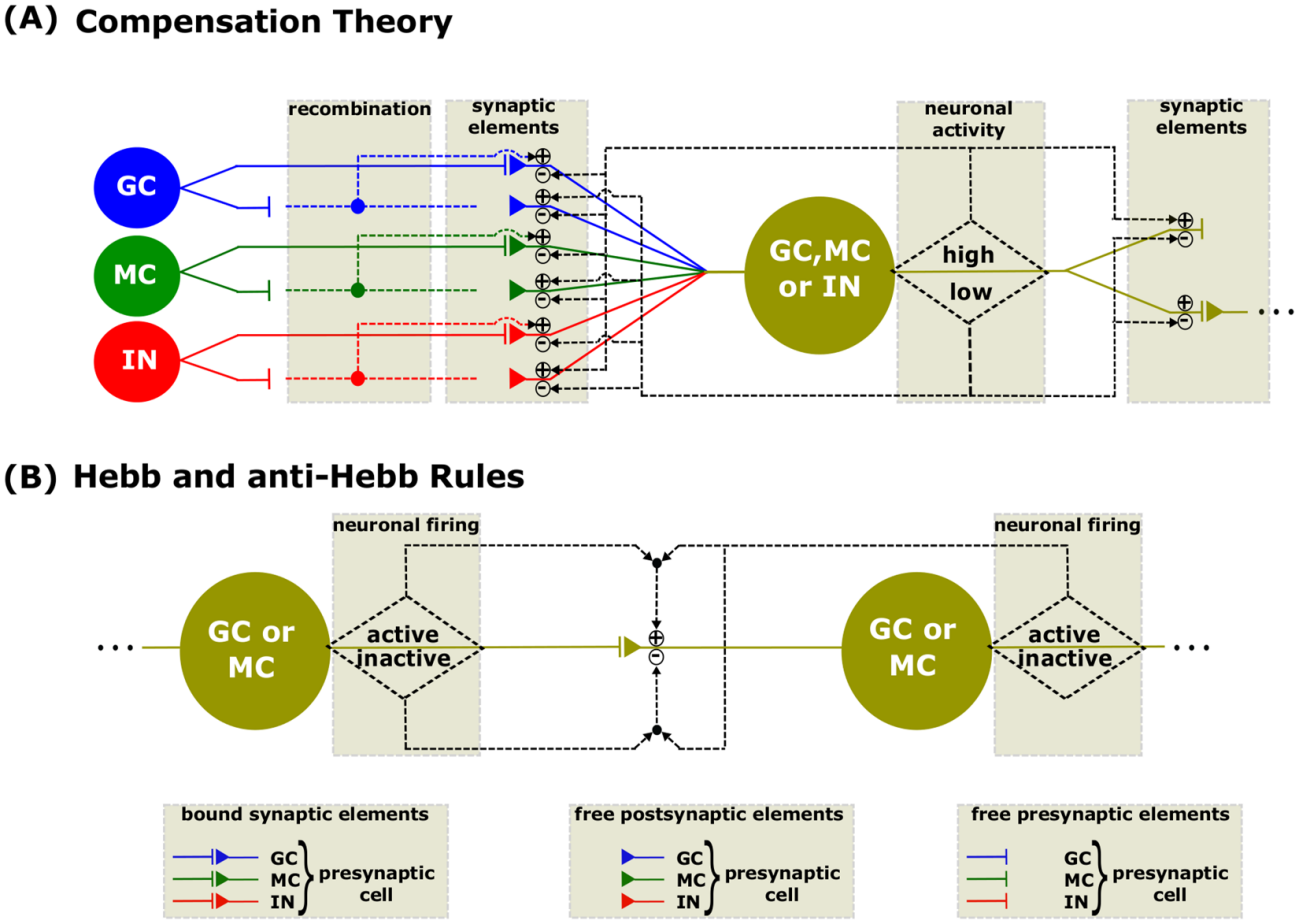
Mechanisms responsible for the change in connectivity of the neural network. (**A**) According to the Theory of Compensation, the synaptic connectivity of the neurons changes as a function of the changes in the level of cellular activity. (**B**) According to the Hebb learning rule, a synapse is enhanced when the firing of the presynaptic cell contributes to the firing of the postsynaptic cell. On the other hand, in anti-Hebb plasticity, the synapses are depressed when the postsynaptic cells fire without contribution from the presynaptic cell.

**Figure 3:**
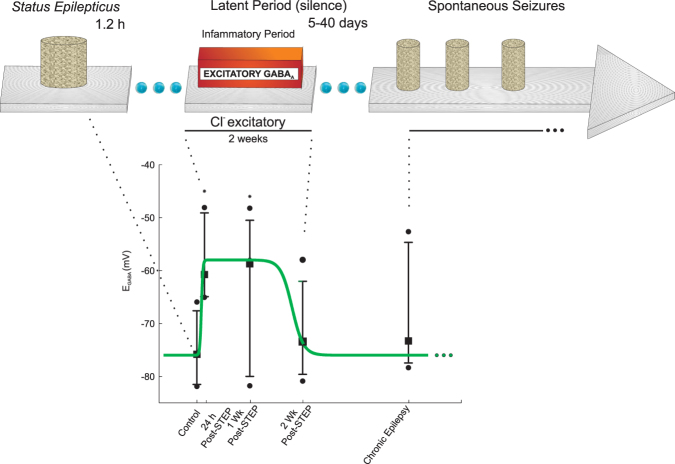
Procedure adopted to simulate the inversion of the effect of GABA_a_ interneurons, from inhibitory to excitatory. The dots are experimental records extracted from Pathak *et al*.^11^ that were used to adjust equation 1, which represent the weight of inhibitory synapses that are changing with time, related to the granule cells with transient excitatory GABA_a_.

To investigate the possible changes in the network synaptic connectivity in the course of the installation of the spontaneous epileptiform activity after *SE*, we performed computational simulations. Using the formalism of McCulloch-Pitts^15^, the neuronal network was based on the mutual interaction between three populations of neurons: granule cells (GC), mossy cells (MC) and interneurons (IN) (Fig. 1A). The changes in the connections between the neurons were based on compensation theory and the Hebbian and anti-Hebbian rules, which are both dependent the on neuronal activity^10,16,17^ (Figs 1B and 2). The level of activity of each cellular type was described according to the McCulloch-Pitts formalism as well as in dependence on the non-synaptic mechanisms. Simulations that represented an intense depolarization of several neurons were performed to represent an experimental protocol (Fig. 3) in which a stimulus, such as pilocarpine, is used to induce *SE*.

During the inflammatory period that follows *SE*, part of the granule cell population was submitted to an inversion in their response to GABAa (being excited instead of inhibited), which happens during the inflammatory period. According to Pathak *et al*.^11^, the inflammatory period lasts for approximately 2 weeks. The changes in the connectivity, induced during this period, contribute significantly for the occurrence of spontaneous seizures.

**Figure 4:**
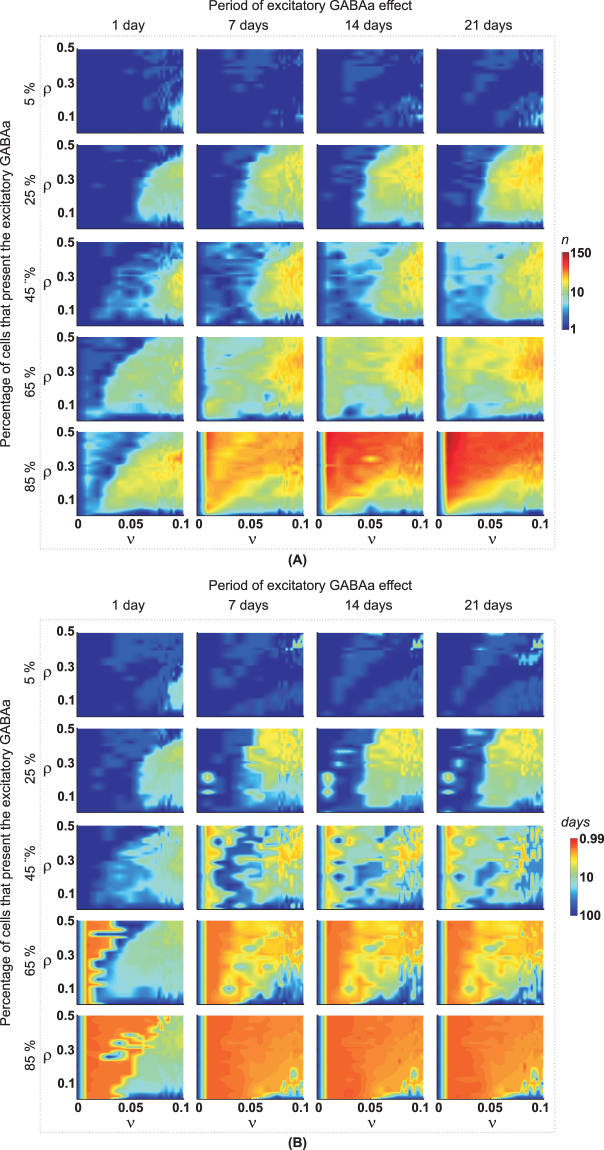
The occurrence of (**A**) epileptiform activity and (**B**) latency for the appearance of the first epileptiform activity, in terms of number of cells that present the transient excitatory GABA_a_ and number of days of the excitatory GABA_a_ effect, respectively, as a function of different combinations of Compensation Theory (ν) and Hebbian Rule (ρ). The amount of epileptiform activity was determined within 100 days after *SE*.

When a few granule cells exhibited an excitatory GABAa response, the increase in the duration of the excitatory GABAa after *SE* did not show a significant effect on the latency and the occurrence of epileptiform activity (Fig. 4). This finding occurred for all combinations of the rate parameters, ν and ρ, which are associated, respectively, with the Compensation Theory and the Hebbian rules. Only when the number of granule cells that exhibited the excitatory GABAa response exceeded 25% did the increase in the duration of the excitatory GABAa intensify and reduce the latency for the spontaneous increase in the cellular activity. We also observed an expansion of the red colour region of ν × ρ, which is associated with spontaneous epileptiform activity.

**Figure 5:**
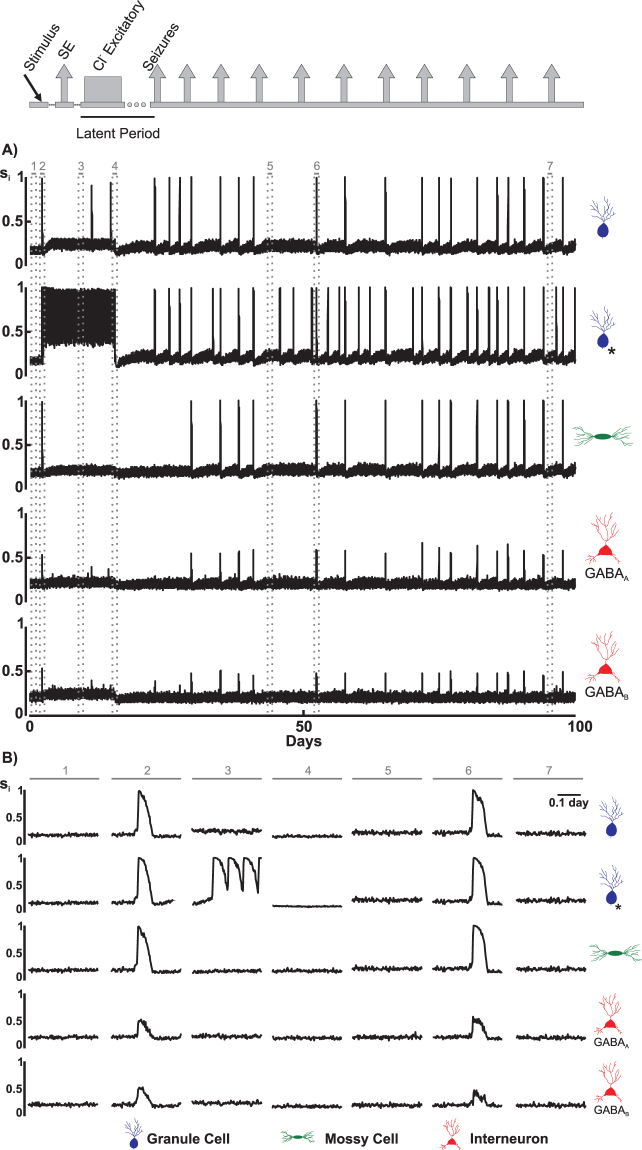
(**A**) Cellular Activity (*s*_*i*_) of the different types of DG cells during the simulation of the induction process of epileptiform activities for ν = 0.1 (Compensation Theory) and ρ = 0.3 (Hebbian Rules). (*) indicates a granule cell that underwent inversion of the effect of GABA_a_, from inhibitory to excitatory. (**B**) Samples of activities for the different cell types, showing 7 moments: (1) rest, (2) *SE*, (3) excitatory GABA_a_, (4) silence, (5 and 7) interval between epileptiform activity and (6) epileptiform discharge.

To inspect how variations in the synaptic connectivity would be associated with changes in the level of cellular activity, we performed simulations with ν = 0.1 and ρ = 0.3, which correspond to the case in which there was a higher occurrence of epileptiform activity with lower latency. For these simulations, we also assumed that 25% of the granule cells exhibited excitatory GABAa for 14 days, values that were estimated experimentally^11,18,19^. Figure 5 shows the evolution of the level of cellular activity of the different types of cells in the neuronal network. Stimulation of the neuronal network at 1.5 days increases the neuronal activity, which represents the *SE* (Fig. 5 – interval 2), with a duration of ~40 min. Later, during the period in which the GABAa remains excitatory (Fig. 5 – interval 3), an increase in the activity of the granule cells is observed, especially cells whose GABAa effect has been inverted. Immediately after the period of the excitatory GABAa, there is a reduction in the activity of the cells (Fig. 5 – interval 4), mainly granule cells and interneurons. Subsequently, all of the cellular activity of the neuronal network increases progressively. Even between subsequent increases in the cellular activity (such as intervals 5 and 7, Fig. 5), the activity remains higher than that observed before the *SE*. In addition, after each spontaneous increase in activity, the level of activity of the neuronal network was reduced. However, the activity was lower than that observed after the end of the excitatory GABAa period.

**Figure 6:**
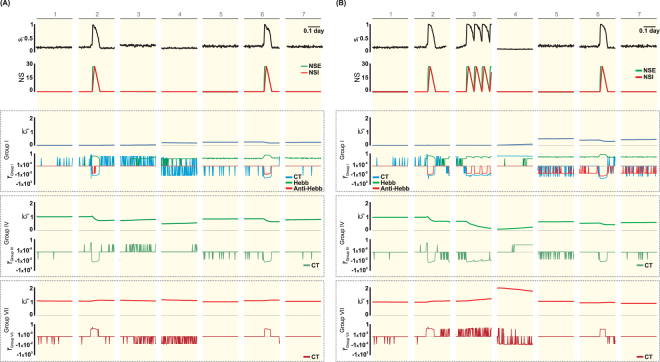
Activity of Granule Cells (*s*_*i*_), non-synaptic mechanisms (NS), connectivity (${\bar{c}}_{ij}$) and rates of connectivity change ($\bar{r}$) for ν = 0.1 (Compensation Theory) and ρ = 0.3 (Hebbian Rules). ${\bar{c}}_{ij}$ and $\bar{r}$ for each connectivity groups (I, IV and VII) are shown, corresponding to the synapses sent to the GC from GC, MC and interneurons. (**A**) Granule cell that does not exhibit transient excitatory GABA_a_. (**B**) Granule cell that presents transient excitatory GABA_a_. The intervals 1–7 are those indicated in Fig. 5: (1) rest, (2) *SE*, (3) excitatory GABA_a_, (4) silence, (5 and 7) interval between epileptiform activity and (6) epileptiform discharge. Symbols: NSE - effect of non-synaptic excitatory mechanisms, NSI - effect of non-synaptic inhibitory mechanisms, CT - Compensation Theory, Hebb - Hebbian Rules, Anti-Hebb - Anti-Hebbian Rules.

In response to stimulation and during spontaneous epileptiform discharges, increased neuronal activity is initiated when the effect of non-synaptic excitatory (NSE) mechanisms outweighs the effect of non-synaptic inhibitory (NSI) mechanisms (NSE > NSI), and it ends when NSI exceeds NSE (Fig. 6A, intervals 2 and 6). For granular cells that did not have GABAa altered from inhibitory to excitatory, during the period of increased activity that represents SE (Fig. 6A, interval 2), significant changes in the connectivity between the granule cells (Group I) were not observed. This finding arises because, although the connectivity changes caused by the Hebbian rule (${\bar{r}}_{GroupI}^{Hebb} > 0$, where ${\bar{r}}_{Group}^{mr}$ represents the rate of connectivity change due to morphogenetic rules (mr: CT, Hebb or Anti-Hebb) for a given connectivity group: Group I, …, IX) contributes to increasing the connectivity between the granular cells, the connectivity changes by compensation theory (${\bar{r}}_{GroupI}^{CT} < 0$) and anti-Hebbian rule (${\bar{r}}_{GroupI}^{Anti-Hebb} < 0$) contribute to reducing the connectivity (Fig. 6A). Within interval 3 (Fig. 6A), with increased activity due to the indirect effect of excitatory GABAa, a slow formation of group I synapses is observed. It should be noted that both morphogenetic changes, those from compensation theory and from the Hebbian rules, contribute to increasing the connectivity (${\bar{r}}_{GroupI}^{Hebb} > 0$ and ${\bar{r}}_{GroupI}^{CT} > 0$). After the excitatory GABAa period, when the cellular activity is very low (Fig. 6A, interval 4), the connectivity between the granular cells increases. This increase, which is associated with an increase in the connectivity change promoted by Hebbian rules, continues progressively until the onset of spontaneous epileptiform discharges (Fig. 6A, interval 6). During the spontaneous epileptiform discharges, a reduction in the connectivity between the granular cells, which is caused by the compensation theory and by the anti-Hebbian rule, is observed. During the intervals between spontaneous epileptiform discharges (Fig. 6A, intervals 5 and 7), the effect of the Hebbian rule predominates, causing a progressive increase in the interconnection between the granular cells. With respect to the connections that the mossy cells sent to the granular cells (group IV), a reduction caused by the compensation theory (${\bar{r}}_{GroupIV}^{CT} < 0$) is observed in response to the increase in the granular cell activity. As can be observed during the increase in the activity, either stimulus-induced (Fig. 6A, interval 2) or spontaneous (Fig. 6A, interval 6), the more intense the neuronal activity is, the greater the decay of the connectivity of this connection group. In the case of interneurons inhibiting granular cells (group VII), the changes caused by compensation theory were intensification (${\bar{r}}_{GroupVII}^{CT} > 0$) during an increase in activity and reduction (${\bar{r}}_{GroupVII}^{CT} < 0$) during periods with a low activity level. The net effect is that the occurrence of epileptiform activity causes an increase in the connectivity through this type of connection.

According to the simulations, the granule cells in which the effect of GABAa was transiently inverted (Fig. 6B) exhibit more intense alterations in the level of activity and in their connectivity in the neuronal network when compared to the other granule cells (Fig. 6A). This difference is due to the high level of activity of the former group of cells during the period of excitatory GABAa (Fig. 6B, interval 3).

**Figure 7:**
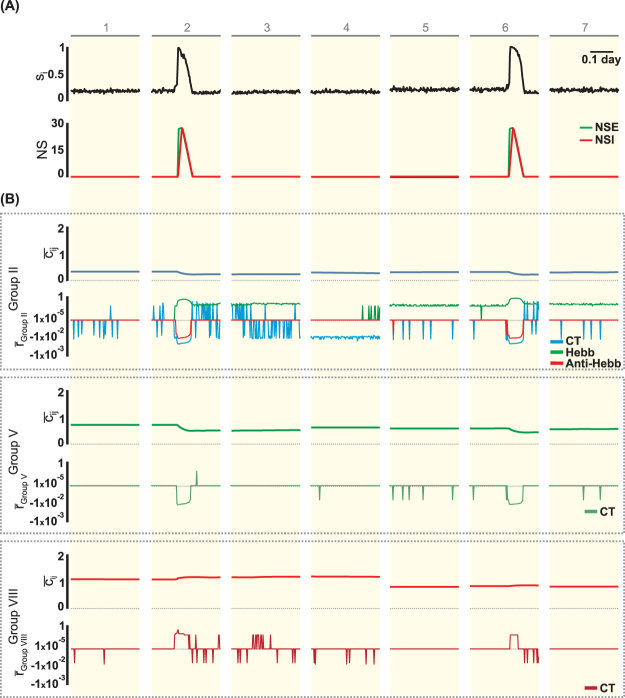
Activity of MC (*s*_*i*_), non-synaptic mechanisms (NS), connectivity (${\bar{c}}_{ij}$) and rates of connectivity change ($\bar{r}$) for ν = 0.1 (Compensation Theory) and ρ = 0.3 (Hebbian Rules). ${\bar{c}}_{ij}$ and $\bar{r}$ for each connectivity group (II, V and VIII) are shown, corresponding to synapse sent to the MC from GC, MC and interneurons. The intervals 1–7 are those indicated in Fig. 5: (1) rest, (2) *SE*, (3) excitatory GABA_a_, (4) silence, (5 and 7) interval between epileptiform activity and (6) epileptiform activity. Symbols: NSE - effect of non-synaptic excitatory mechanisms, NSI - effect of non-synaptic inhibitory mechanisms, CT - Compensation Theory, Hebb - Hebbian Rules, Anti-Hebb - Anti-Hebbian Rules.

When accounting for the mossy cells, the simulations suggest that the increase in the neuronal network activity, the effect of the SE and of the consequent spontaneous depolarization (Fig. 7, interval 6) lead to a reduction in the excitatory connections from the granule and mossy cells (Groups II and V). This change occurs due to the effect of the compensation theory (${\bar{r}}_{GroupII}^{CT} < 0$ and ${\bar{r}}_{GroupV}^{CT} < 0$), which guides a reduction of the most intense synapses during the intervals in which the activity increases, thus acting as a control of the level of activity of the neuronal network. With the same objective of controlling the increase in the activity of the mossy cells, the compensation theory causes the formation of more inhibitory synapses (${\bar{r}}_{GroupVIII}^{CT} > 0$), thus increasing the inhibitory effect of the interneurons on these excitatory cells. At intervals at which the level of mossy cell activities is lower (Fig. 7, intervals 3–5 and 7), the compensation theory contributes to the connectivity at the input of the mossy cells (excitatory and inhibitory) to return to the normal level (${\bar{r}}_{GroupII}^{CT} > 0$,${\bar{r}}_{GroupV}^{CT} > 0$ and ${\bar{r}}_{GroupVII}^{CT} < 0$).

**Figure 8:**
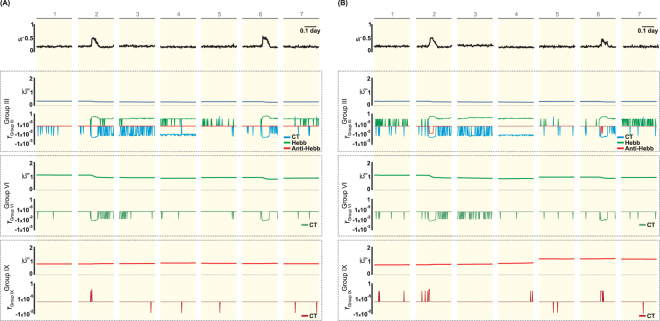
Activity of interneurons (*s*_*i*_), connectivity (${\bar{c}}_{ij}$) and rates of connectivity change ($\bar{r}$) for ν = 0.1 (Compensation Theory) and ρ = 0.3 (Hebbian Rules). ${\bar{c}}_{ij}$ and $\bar{r}$ are shown for each connectivity groups (III, VI and IX), corresponding to synapses sent to interneurons from, respectively, GC, MC and interneurons. (**A**) Interneuron GABA_a_. (**B**) Interneuron GABA_b_. The intervals 1–7 are those indicated in Fig. 5: (1) rest, (2) *SE*, (3) excitatory GABA_a_, (4) silence, (5 and 7) interval between epileptiform activity and (6) epileptiform activity. Symbols: CT - Theory of Compensation, Hebb - Hebbian Rules, Anti-Hebb - Anti-Hebbian Rules.

With regard to the interneurons, during the activity increase (Fig. 8, intervals 2 and 6), there is a reduction in the excitatory synaptic inputs from granule and mossy cells (Groups III and VI, respectively) as a result of the effect of the synaptic decay caused by the compensation theory (${\bar{r}}_{GroupIII}^{CT} < 0$ and ${\bar{r}}_{GroupVI}^{CT} < 0$). The inhibition of GABAa-like interneurons (Fig. 8A) did not change significantly. However, in the case of GABAb-type interneurons (Fig. 8B), there was an increase in the inhibition between the interneurons (Group IX), which is an effect of the theory of compensation (${\bar{r}}_{GroupIX}^{CT} > 0$).

**Figure 9:**
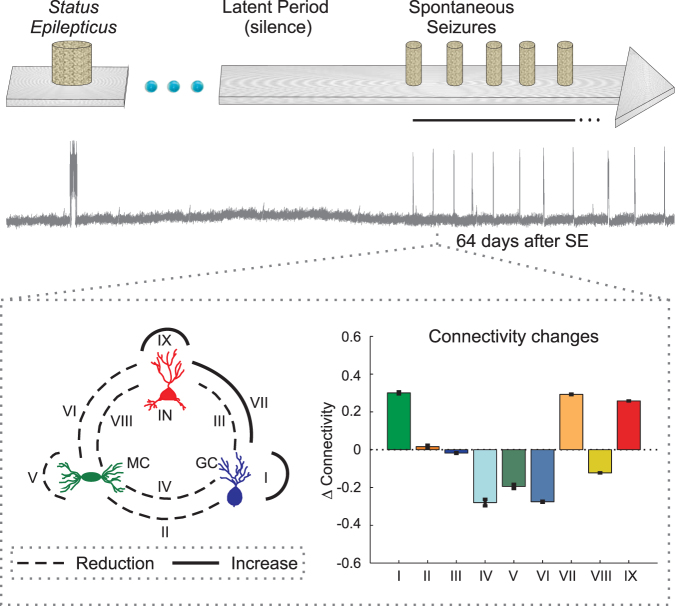
Connectivity changes of the different groups of connections of the DG network for ν = 0.1 (Compensation Theory) and ρ = 0.3 (Hebbian Rules). The connectivity changes were measured in relation to the mean number of connections of each group (I–IX) observed before *SE* induction. Variations were calculated 64 days after *SE*, after the appearance of the epileptiform activities, as shown in the above diagram. From this period, the connectivity baseline, during intervals between epileptiform discharges, does not show large changes. Data presented as the mean ± SEM (n = 12).

The main changes in the connectivity of the neuronal network of the DG due to the process of epileptiform activity induction are as follows (Fig. 9): (i) the increasing connectivity between granular cells (group I), which represents the abnormal sprouting of mossy fibres (ASMF); (ii) the reduction of the connectivity of mossy cells within the DG circuitry, both input and output synapses (groups II, IV–VI, VIII); and (iii) the inhibition of GABAb interneurons (group IX).

**Figure 10:**
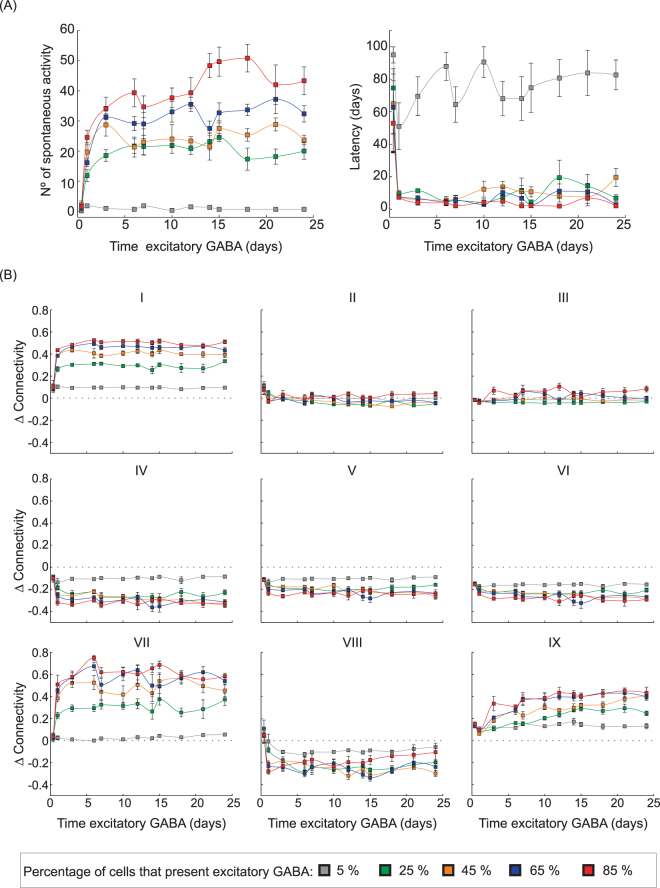
(**A**) Effects of the number of granule cells presenting excitatory GABAa and the effect of transient excitatory GABA_a_ on the occurrence of epileptiform activity (Left) and on the latency period for the occurrence of epileptiform activity (Right). (**B**) Network connectivity changes for the different groups. The number of epileptiform discharges was counted along 100 days after the *SE*, and the latency corresponds to the time for the first epileptiform activity appearance. The connectivity changes were calculated, for each connection group, 64 days after *SE* and in relation to the average connections before *SE* induction. Simulations were performed for the worse case: ν = 0.1 (Compensation Theory) and ρ = 0.3 (Hebbian Rules). The data are presented as the mean ± SEM (n = 12).

Analysing the effect of the period of GABAa inversion (inhibitory to excitatory), simulations were performed by varying the time that the GABAa remains transiently excitatory and the number of granular cells that exhibit excitatory GABAa (Fig. 10). In Fig. 10A, when few granular cells exhibit the transient excitatory GABAa (5%), the time that the GABAa remains transiently excitatory has no significant effect on the occurrence of epileptiform discharges, latency, and on the connectivity changes. The increase in the number of granule cells that exhibit excitatory GABAa (25, 45, 65 and 85%) causes a higher occurrence of epileptiform activity and intensifies the changes in the connectivity, as follows (Fig. 10B): (i) an increased connectivity between granular cells (Group I); (ii) a reduced connectivity of mossy cells (Groups II, IV–VI and VIII); and (iii) an increased inhibition of granule cells (Group VII) and between interneurons (Group IX). The simulations also show that the occurrence of epileptiform discharges and that the connectivity changes saturate when GABAa remains excitatory for more than 5 days.

**Figure 11:**
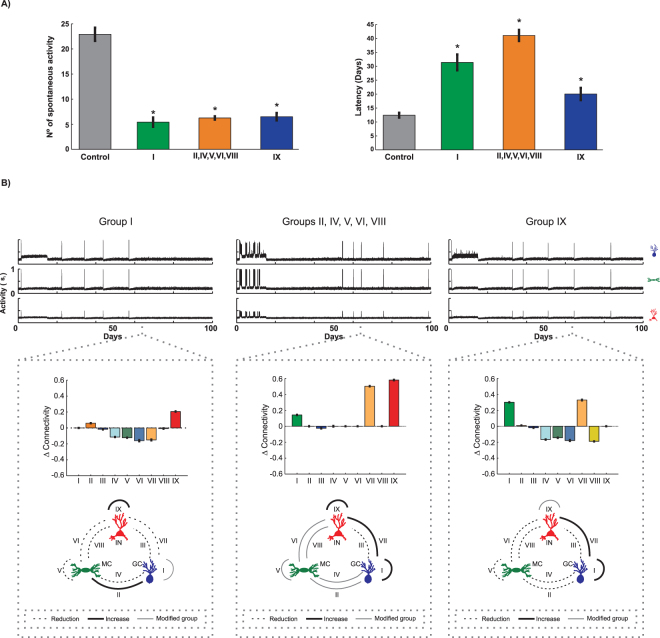
(**A**) Number of spontaneous activities (Left) and latency period (Right) for the beginning of activities for the Control group and for the withdrawal of the connectivity changes of the groups I; II, IV, V, VI, VIII, simultaneously; and IX. (**B**) Activity and connectivity changes for each connection group considered 64 days post-*SE*. The scheme showing the circuitry represents the changes that occurred with each connectivity group 64 days post-*SE* and in relation to the mean value of the connectivity before the induction of the *SE*. Simulations were performed for ν = 0.1 (Compensation Theory) and ρ = 0.3 (Hebbian Rules). The data are presented as the mean ± SEM (*p < 0.05) (n = 12).

Intensified by the excitatory GABAa, the connectivity changes that affect the process of spontaneous epileptiform discharges induction were investigated. It was observed that the simulation of the blockage of these changes (Group I or Groups II, IV–VI and VIII or Group IX) suggest a reduction in the number of spontaneous neuronal discharges and an increase in the latency of the epileptiform activity onset (Fig. 11A). Analysing the effect of this blockage on the connectivity changes of Group I (Fig. 11B) in comparison with the control simulations (Fig. 9), less intense changes occur in connections in which the mossy cells are pre-synaptic (Groups IV–II) as well as having less intense connectivities among the interneurons (Group IX). Moreover, different from the control simulations, Group II exhibited a connectivity increase and a reduction in the inhibition of the granule cells (Group VII). In this simulation, although there are still connectivity changes due to SE and transient excitatory GABAa, the connectivities of Groups II–IX showed a tendency to be restored with temporal evolution. The suppression of the connectivity changes of Groups II, IV, V, and VI (Fig. 11B), which are the connection of the mossy cells of the neuronal network, reduced the formation of synapses between the granule cells (Group I) and increased the inhibition of these cells (Group VII) and of the interneurons (Group IX). When the connectivity changes of Group IX (inhibition between interneurons) are blocked, although there is an increase in the connectivity between the granule cells (Group I), the connectivity of groups IV–VI (Fig. 11B) exhibited smaller reductions in relation to the control simulation, which indicates greater preservation of the synaptic connections of mossy cells with the neuronal network. In addition, the inhibition of granule cells by interneurons (group VII) increased, similar to the control simulations.

## Discussion

To investigate the mechanisms that are involved in the changes of the synaptic connections of the DG in dependence on the level of the neuronal activity during the induction of epileptiform activity, we performed computational simulations. The simulations show that after status epilepticus (SE), during the latent period, some of the granular cells underwent inversion of the GABA_a_ effect (from inhibitory to excitatory), due to intracellular accumulation of Cl^−^, thus increasing their excitability. Under this circumstance, the whole network sustains synaptogenesis. In the model, the synaptogenic processes were governed by compensation theory and Hebbian and anti-Hebbian rules. These processes result in the formation of new GABAergic synapses to control the seizure. However, these synapses act by exciting the post-synaptic cells instead of inhibiting them, which acts as positive feedback, thus inducing more excitation. After the [Cl^−^]_i_ recovery, which was described by Pattak *et al*.^11^, the GABAa inhibition returns under a scenario of increased GABAergic synapses. Therefore, an intense hyper-polarization of the granule cells occurs. To compensate the hyper-polarization state, the granule cells increase their collateral formation of aberrant mossy fibre sprouting. To substantiate this hypothesis, the simulations show neuronal activities that resemble experimental observations that offer possible mechanisms. Conjointly with the abnormal sprouting of the mossy fibres, the simulations show a reduction in the mossy cell connections in the network, which can be associated with findings that show the vulnerability of the mossy cells in temporal lobe epilepsy^12^. The simulations also show concomitantly increased inhibition between the interneurons, which could be an alternative to the dormant basket cell hypothesis^13,14^ and suggests a possible mechanism for the disinhibition of the granule cells.

After the *SE* induction, morphofunctional changes are observed in the brain tissue during a period called the latent period, when spontaneous seizures are not disrupted^8^. Only after the latent period do spontaneous epileptiform seizures occur. In this type of experimental protocol, immediately after the *SE*, a period called the inflammatory period is observed, where the GABA_A_ effect changes from inhibitory to excitatory due to an intracellular accumulation of Cl^−^^11,20,21^. Investigating the excitatory GABA_A_ effect during the process of epileptiform activity induction, the simulations reinforce the hypothesis that the abnormal mossy fibre sprouting contributes to epileptogenesis. Moreover, the simulations allowed for the proposal that the reduction in the connectivity of the mossy cells in the synaptic network of the DG and the increase of the inhibition between the interneurons could also contribute to the epileptiform activity.

Clinical and experimental evidence indicates that disturbances in the GABA_A_ signalling could facilitate the seizures^22^ since this reduction in the synaptic regulation would induce a disequilibrium between excitation and inhibition^1,3,23^, thus compromising the inhibition and potentially contributing to the epileptogenesis^24–26^. The simulations indicate that the disequilibrium, even if transitory and persisting for hours, can disrupt structural synaptic changes in the network, which evolves to spontaneous epileptiform activity (Figs 5 and 10). During the period in which the GABAa remains excitatory, the main observation is the increase in the activity of the network, especially of the cells in which the inversion of the GABAa effect occurred (Fig. 5). During this period, an increase in the rate of inhibitory synapse formation occurs in granule cells (Fig. 6). Therefore, when the excitatory GABAa period ends, the level of cellular activity falls below the resting level (Fig. 4), which favours the formation of new excitatory synaptic elements by compensation theory, and these elements are substrates for synaptic reinforcement, according to the Hebbian rule. Thus, excitatory GABAa causes changes in the synaptic network that favour the onset of epileptiform discharges (represented by spontaneous increases in the activity). In addition, these changes are intensified when the period of the excitatory GABA_A_ increases and/or the number of cells in which excitatory GABA has occurred increases (Fig. 10).

As is known, abnormal sprouting of mossy fibres (ASMF) is associated with epilepsy in humans and is also present in animal models of epilepsy. According to the simulations, among the changes in the synaptic network that contribute to the epileptiform activity, the increase in connectivity between the granule cells (Group I) could be the result of a combination of compensatory effects, as described by Compensation Theory and the synaptic reinforcement described by Hebbian and anti-Hebbian Rules^10^. Describing this mechanism after more intense depolarizations, such as those that occur during *SE* and especially during the excitatory GABA_A_ effect, a period of low cellular activity is observed in which new excitatory synapses are formed, according to Compensation Theory, thus interconnecting the granule cells and characterizing the ASMF. Once these cells are interconnected, the synaptic reinforcement occurs according to Hebbian Rules, and consequently, the ASMF is enhanced. Giving support to this hypothesis, an increase in neurogenesis in the DG has been observed during the epileptogenic period^27–29^. Moreover, younger granular cells have a high capacity for synaptic plasticity and a higher propensity for structural abnormalities, including ASMF^30,31^. In the simulations performed in the present work, the younger granule cells can be interpreted as being those that present higher values of Compensation Theory (υ) and Hebbian and anti-Hebbian Rules (ρ), and in this case, an increase in the cell age corresponds to the reduction of υ and ρ. It can be observed that as υ and ρ decrease, in other words, as the cells become older, the occurrence of epileptiform activity becomes lower (Fig. 4); in addition, there is a less intense increase in the connectivity of Group I, in other words, the ASMF is less intense (data not shown). Supporting this hypothesis, according to Kron *et al*.^32^ and Hester and Danzer^31^, the granule cells become insensitive to the structural changes caused by the process of epileptogenesis when they mature.

Although the effect of ASMF on the seizure-inducing process is still not well understood^33^, there are studies that suggest that the ASMF contributes to the generation of and sustaining epileptiform activity^34–37^. Supporting this hypothesis, the simulations of the present work suggest that the increase in the granule cell activity that results from ASMF contributes to the onset of epileptiform activity. As seen in Fig. 11, when the changes in the connectivity of Group I (GC =  > GC) are blocked, the occurrence of epileptiform activity decreases. In addition to the effect on the level of activity, the simulations also suggest that ASMF contributes to intensifying the synaptic changes of the DG since the blockage of the changes of the connectivity of Group I reduced the connectivity changes of the other groups of connections.

According to the simulations, other changes in the intrinsic synaptic network that contribute to the appearance of epileptiform activity are the reductions in the connectivity of Groups IV–VI and VIII (Figs 10 and 11), which represent the reduction of the contribution of mossy cells to the level of activity of DG (Group IV–VI) and the control of the activity level of these cells by the interneurons (Group VIII). In addition, the results suggest that changes in the synaptic structure contribute to an increase of the activity of mossy cells, and as a result, the mossy cells are prone to cell death. One of the hypotheses that explain the DG participation in the generation of and sustaining epileptiform activity is the dormant basket cell theory^12,13^: the death of mossy cells, which occurs during the process of epileptogenesis, leads to a lower activation of interneurons and, consequently, to the disinhibition of granular cells. The simulations of the present work suggest that it is not necessary that the death of the mossy cells occurs in such a way that the inhibition of the interneurons on the granule cells decreases. Even with mossy cells exhibit a higher level of activity, the reduction of the connectivity of the mossy cells within the synaptic network of DG is sufficient to decrease the activation of the interneurons and contribute to the disinhibition of the granule cells and, therefore, to the appearance of the epileptiform activity. According to the simulations, increased connectivity among the interneurons (Group IX) also contributes to the development of epileptiform activity (Figs 10 and 11). In this case, one interneuron inhibits the other and, consequently, causes the disinhibition of granule cells and favours the onset of seizures. This mechanism is another mechanism that could contribute to the theory of dormant basket cells. Anatomic and electrophysiological studies provide evidence that GABAergic cells in the hippocampal formation could innervate one another, and the interneurons can thus be controlled by specific inhibitory mechanisms^38–40^.

## Conclusions

The changes in the synaptic network that occur during the process of epileptogenesis are complex and could contribute to the onset of epileptiform activity. In the present work, we use mathematical models and computational simulations that describe changes in the synaptic network (Compensation Theory and Hebbian and Anti-Hebbian Rules) that are dependent on the level of network activity. Considering only these processes of synaptic alteration, the simulations allow us to propose that in the post SE period, the inversion of the GABA_A_ effect, from inhibitory to excitatory, contributes to the onset of epileptiform activities, mainly by intensifying the excitatory synaptic interconnections between GC, which describes the ASMF; by causing inhibition between INs; and by reducing the connectivity of mossy cells within the synaptic network of DG.

## Methods

The mathematical model, whose detailed description is presented in the appendix (supplementary information), represents the synaptic network of the DG of the rat hippocampus^10^. Assuming the three main neurons of the DG (GC, MC and interneurons) as the three cell types of the network, nine possible groups of synaptic connectivity were represented: groups I–IX (Fig. 1A). The groups I, II and III represent the excitatory synaptic connections where the GC are pre-synaptic and the pos-synaptic are, respectively, GC, MC and interneurons. For the groups IV, V and VI, the MC send, respectively, excitatory synapses to GC, MC and interneurons. The groups VII, VIII and IX represent inhibitory synapses sent, respectively, from interneurons to GC, MC and interneurons. All synapses of the neuronal network were represented by a connectivity matrix C_NxN_, where element *c*_*i,j*_ represents the synaptic connectivity, *j* is the pre-synaptic neuron and *i* the pos-synpatic (supplementary information - Equation A.1).

As represented in Fig. 1B and described by equations A.2–A.11 (supplementary information), the intensities of the synaptic connectivity and the neuronal firing of the pre-synaptic neurons have direct effect on the membrane potential of each pos-synaptic neuron. Moreover, the membrane potential is also dependent on the non-synaptic mechanism effects, which may be more effective during the ictal period of the epileptiformes activities^41^. In dependence on the membrane potential (Vm), the firing probability is calculated and sorting from a uniform distribution (from 0 to 1) is determined if the neuron will fire (active) or not (inactive). The level of neuronal activity *s*_*i*_^*t*^ is also calculated from the average firing probability during an interval of time. Depending on the value of *s*_*i*_^*t*^, the neuron is considered in resting state (0.15 < *s*_*i*_^*t*^ < 0.25), low level of activity (*s*_*i*_^*t*^ < 0.15) or high level of activity (*s*_*i*_^*t*^ > 0.25).

The level of neuronal activity defines the synaptic connectivity changes for each synaptic group by means of the Compensation Theory and the Hebbian and anti-Hebbian Rules^42–45^ (Fig. 2). According to the Compensation Theory, when the neuron is in resting state, no connectivity changes occur. On the other hand, if the level of neuronal activity is high, reduction of the number of bound excitatory pos-synaptic elements occur and also occur the offer of free excitatory pos-synaptic buttons. The high level of neuronal activity also causes the increase of the offer of free inhibitory pos-synaptic buttons and of free pre-synaptic buttons. When the level of neuronal activity is low, the offer of free excitatory synaptic buttons increases and the bound inhibitory pos-synaptic elements reduces and also reduces the offer of free pre-synaptic elements and of the bound pre-synaptic elements. These changes in the synaptic connectivity governed by the Compensation Theory (Fig. 2A) are described by the equations A.12–A.24 and Table A.1 (supplementary information).

The firing effect of the pre-synaptic neurons contributing for the firing of the pos-synaptic neurons also causes changes in the synaptic connectivity, following the Hebbian Rule^46–48^ (Fig. 2B). In the model, according to this rule, when a pre-synaptic neuron fire (active state) in a given iteration and the pos-synaptic neuron also fire (active state), in the following iteration, it occurs the increase of the synaptic connectivity (supplementary information - Equations A.25 and A.26). On the other hand, when the pre-synaptic neuron does not contribute for the pos-synaptic neuron fire, occurs the reduction of the synaptic connectivity, following the anti-Hebbian Rule^46^ (Fig. 2B). In the model, representing this rule, when the pos-synaptic neuron become active in a given iteration and the pre-synaptic neuron was not active in the preceding iteration, a reduction in the connectivity happens (supplementary information - Equation A.27).

### Computational Simulations and Parameters

Animal models are used to study epilepsy related to chemical agents, such as pilocarpine, or electric stimulation of brain structures, which are used to induce *SE*^49^ (Fig. 3). After *SE*, which can last for tens of minutes to hours, a latent period (absence of seizures) with a duration of one or more weeks occurs. Subsequently, the disruption of spontaneous seizures characterizes the induction of the experimental epilepsy period^50,51^. At the beginning of the latent period, which typically occurs approximately 2 weeks after the inflammatory period, the GABAa equilibrium potential (E_GABA_) remains more positive than the membrane potential. Therefore, instead of inhibition, the effects of the GABAa synaptic receptors on the target neurons are of excitation^1,52–54^. Reproducing this experimental circumstance, the computational neuronal network was stimulated by setting α = 1.0 (Equation A.3) for a duration of 70 min. The stimulus increases the neuronal activity and synchronism (Fig. 5), which characterize the *SE*^10^. As consequence of the synaptogenesis rules described above, a latent period also follows the *SE*. Representing the transitory excitatory GABAa period, at the beginning of the latent period, the inhibitory synapses of the granule cell population that have two of the three interneurons as pre-synaptic neurons were changed to excitatory, according to Equation 1. After the latent period, the neuronal network sustains spontaneous increases of the cellular activity that represent epileptiform activity.

1 $$\varphi =\overline{\varphi }\times (\tanh (\frac{t-{t}_{i}}{0.2})+1.0)\times (\tanh (\frac{{t}_{f}-t}{1.5})+1.0)$$

where $\bar{\phi }$ is a constant, and t_i_ = 2.0 days (0.5 day after the stimulus) represents the beginning of the excitatory GABAa period, where t_f_ represents the end of the period. This equation was adjusted to represent the average behaviour of the E_GABA_ that was experimentally recorded (Fig. 3). According to Pathak *et al*.^11^, approximately 22% of the granule cells exhibit transitory excitatory GABAa after *SE*, and this effect can last up to 2 weeks. For cells in which the GABAa inversion is absent, the weight of the inhibitory synapses is given by

2 $$\varphi =\overline{\varphi }$$

In the present work, how the number of granule cells, with excitatory GABAa responses and the time duration of the excitatory GABAa, affect the GD synaptic circuitry changes and, consequently, the epileptiform activity induction was investigated. The combinations of different numbers of excitatory GABAa granule cells, {5, 25, 45, 65, 85} %, with different durations of the GABAa inversion, {1, 7, 14, 21} days, were investigated. For each of these combinations, we performed simulations with different values of the rates ν and ρ, respectively, related to compensation theory and Hebbian and anti-Hebbbian rules. Here, ν was changed from 0 to 0.1, and ρ was changed from 0 to 0.5. The upper limit of ν (0.1) and ρ (0.5) were determined to guarantee smooth convergence in the changes of the synaptic elements^47^.

The connectivity matrix was initialized considering *c*_*i,j*_ = 0 for 1 ≤ i, j ≤ NE-3 (Group I – Fig. 1), which represents an absence of connections between granule cells under normal conditions. For the remaining connections (Groups II–IX), the initial values of *c*_*i,j*_ followed a normal distribution with an average and standard deviation of 1.0 ± 0.2.

To perform the simulations, two timescales were necessary. One timescale was for the cellular activity changes, which are associated with the duration of each numerical iteration. The other timescale was for the connectivity changes, which correspond to one morphogenetic time step (Δ*s*_*i*_), which represents the spiking remodelling and, similarly, the axonal ramification that connects the postsynaptic targets^47,48^ (supplementary information- Equation A.4). In the model, each morphogenetic time step demands 150 interactions, and each iteration is 0.36 min.

The remaining constants of the model are shown in Table A.2 (supplementary information). The parameters of the equations for calculating the firing probability (Equations 1 and A.3) and the weights of the different groups of synaptic connections (Equation A.5) were adjusted to maintain stability in the network in the interval 0.15 ≤ *s*_*i*_ ≤ 0.25, when no stimulus is applied. Assuming that the threshold for the actuation of the non-synaptic mechanisms, L, is equal to 0.32, the parameters of the equations that describe the non-synaptic mechanisms (supplementary information - Equations A.6–A.11) were adjusted to provide depolarization and repolarization during an ictal period of epileptiform activity, which represents the influx of Na^+^ through channels and efflux by the pump.

For the computational implementation, we used finite difference methods with a tolerance error of 10^−4^. The model was implemented in FORTRAN 90, and a high-performance computer was used (Cluster SGI UV 2000, 80 Cores—Intel Xeon E5-4650v2 10- core, 2.4 GHz, 25 MB Cache, RAM 1024 GB DDR3 1866 MHz, 80 TB HD, SUSE Linux Enterprise Server 11, SGI Performance Suite, Intel Cluster Studio XE).

### Statistical Analysis

In the simulations, a draw was performed in each iteration and for each neuron of the network as a function of the probability of firing, assuming a uniform distribution between 0 and 1, to decide if a neuron will (*z*_*i*_^*t*^ = 1) or not (*z*_*i*_^*t*^ = 0) fire. Therefore, for the same parameters, two simulations may exhibit different results. Therefore, for each investigation (changing of parameters), it was necessary to conduct more than one simulation. In fact, to obtain parametric data, we needed to generate at least 12 repetitions for each parameter combination. Therefore, the results were presented in terms of the mean ± standard error of the mean (SEM). The statistical significance of the comparative analysis was performed using one-way ANOVA followed by Dunnett multiple comparison (p < 0.05).

## Electronic supplementary material

Appendix - Mathematical Model
