# Supplementary material for: GABAa excitation and synaptogenesis after Status Epilepticus – A computational study
Source: Sci Rep. 2018 Mar 8;8:4193. doi: 10.1038/s41598-018-22581-6 (PMC5843660; doi:10.1038/s41598-018-22581-6)
Supplement: Supplementary file 1 — Appendix - Mathematical Model [file 41598_2018_22581_MOESM1_ESM.pdf]

APPENDIX

$GABA_a$ excitation and synaptogenesis after *Status Epilepticus* – A computational study

Keite Lira de Almeida França[1], Antônio-Carlos Guimarães de Almeida[1], Stephen E. Saddow[2], Luiz Eduardo Canton Santos[1], Carla Alessandra Scorza[3], Fulvio Alexandre Scorza[3], Antônio Márcio Rodrigues[1*]

[1] Laboratório de Neurociência Experimental e Computacional (LANEC), Departamento de Engenharia de Biossistemas, Universidade Federal de São João del-Rei (UFSJ), Brazil

[2] Electrical Engineering Department, University of South of Florida, Tampa, FL, USA

[3] Disciplina de Neurologia Experimental, Escola Paulista de Medicina, Unifesp, Brazil

Corresponding Author

Correspondence to

Antônio Márcio Rodrigues

e-mail: amr@ufsj.edu.br

LANEC/UFSJ

Pr. Dom Helvécio, 74

São João del-Rei-MG – 36301-160

BRAZIL

Mathematical Model

The neural network was constructed considering N neurons, where NE <N is the number of excitatory neurons, and N-NE corresponds to the inhibitory cells. Excitatory neurons were divided into {1, ..., NE-3} granule cells (GC), and {NE - 2, ..., NE} hilar mossy cells (MC), whereas inhibitory neurons, {NE + 1, ..., N}, are represented by the interneurons (IN)[10]. The connection matrix between the neurons is given by C (N × N):

$$C = \begin{pmatrix}
c_{1,1} & \cdots & c_{1,NE-3} & c_{1,NE-2} & \cdots & c_{1,NE} & c_{1,NE+1} & \cdots & c_{1,N} \\
\vdots & \cdots & \vdots & \vdots & \cdots & \vdots & \vdots & \cdots & \vdots \\
c_{NE-3,1} & \cdots & c_{NE-3,NE-3} & c_{NE-3,NE-2} & \cdots & c_{NE-3,NE} & c_{NE-3,NE+1} & \cdots & c_{NE-3,N} \\
c_{NE-2,1} & \cdots & c_{NE-2,NE-3} & c_{NE-2,NE-2} & \cdots & c_{NE-2,NE} & c_{NE-2,NE+1} & \cdots & c_{NE-2,N} \\
\vdots & \cdots & \vdots & \vdots & \cdots & \vdots & \vdots & \cdots & \vdots \\
c_{NE,1} & \cdots & c_{NE,NE-3} & c_{NE,NE-2} & \cdots & c_{NE,NE} & c_{NE,NE+1} & \cdots & c_{NE,N} \\
c_{NE+1,1} & \cdots & c_{NE+1,NE-3} & c_{NE+1,NE-2} & \cdots & c_{NE+1,NE} & c_{NE,NE+1} & & c_{NE+1,N} \\
\vdots & \cdots & \vdots & \vdots & \cdots & \vdots & \vdots & \cdots & \vdots \\
c_{N,1} & \cdots & c_{N,NE-3} & c_{N,NE-2} & \cdots & c_{N,NE} & c_{N,NE+1} & \cdots & c_{N,N}
\end{pmatrix}, \qquad \text{(A.1)}$$

where $c_{i,j}$ $1 \le i,j \le N$ is the connection weight from neuron j to neuron i.

The set of states that each neuron of the network can occupy at time t is represented by

$$z^t = (z_1^t, \ldots, z_N^t), \qquad \text{(A.2)}$$

where $z_i^t \in \{0,1\}$, $1 \le i \le N$, t = 0,1,2,... A neuron was considered to be "active" ($z_j^t = 1$) when its membrane potential (Vm) overcomes a specific threshold. When the Vm was lower than the threshold, the neuron was considered to be "inactive" ($z_j^t = 0$).The threshold was estimated by the probability of the neuron to trigger an action potential:

$$prob(z_i^{t+1}) = \frac{1}{1+e^{\frac{MP_i^t+\alpha-\theta}{-\beta}}}, \quad (A.3)$$

where Vm_i^t corresponds to the transmembrane potential, θ is the firing threshold of the cell, β describes the noise of the threshold function and α is a percentage of afferents added to the model to simulate the external perturbations.

The probability $prob(z_i^{t+1})$ was used to determine the mean activity of each neuron during the interval Δt and, consequently, the deviation of each neuron from a desired mean activity, which is called the morphogenetic state of the neuron, Δs_i. This state is calculated for cell i by

$$\Delta s_i = s_i - 0.2, with\, s_i = \frac{\sum_{t_o}^{t_o+\Delta t} prob(z_i^t)}{\Delta t}, \quad (A.4)$$

where t_o, $\Delta t \in \mathbf{N}$, i = 1, ..., N. Here, s_i, which used as a measure of the activity of neuron i, is the average probability of an action potential deflagration.

The Vm was calculated as a function of the inputs from the interconnected cells. Inputs from GCs and MCs contribute to increased Vm, while inputs from INs contribute to a decrease (equation A.5). The Vm is also affected by non-synaptic (NS) effects due to changes in the ionic homeostasis resulting from the intense neuronal activity.

$$MP_i^t = \tau \sum_{j=1}^{NE-3} c_{i,j} z_j^{t-1} + \kappa_u \sum_{j=NE-2}^{NE} c_{i,j} z_j^{t-1} - \phi \sum_{j=NE+1}^{N} c_{i,j} z_j^{t-1} + NS_i^t, \quad (A.5)$$

where τ , κ_u (u = 1 if $i \leq$ NE and u = 2 if $i >$ NE) and φ are the connectivity weights of the synapses in which the pre-synaptic cells j are, respectively, GC, MC and interneurons. τ and κ_u were admitted constant and φ was calculated by means of Equation 1.

NS_i^t is calculated as a function of two terms: excitatory (NSE) and inhibitory (NSI) non-synaptic effects. According to Almeida et al[41], the ictal period of the epileptiform discharges

is sustained by intense changes in the ionic homeostasis during high neuronal firing. During an epileptiform discharge in DG, the intense and synchronized neuronal activity is accompanied by an elevated $[K^+]_o$ that sustains a long depolarization, accompanied by a high influx of Na^+, which is counteracted by efflux of Na^+ through the Na/K pump. The pumping efflux of Na^+ is accelerated by an intracellular Na^+ accumulation. When the Na^+ efflux can overcome the Na^+ influx, the ictal period finalizes. After the ictal period, the neuronal state becomes refractory: first, absolutely refractory, and later, relatively refractory. In this last state, dependent on the network conditions, the neuron can depolarize spontaneously, and the mutual coupling of the whole network will sustain a recruited depolarization that characterizes another ictal period. Therefore, in the present model, NSE represents the Na^+ influx and NSI the Na^+ efflux through the pump. NS, the difference between NSE and NSI, resembles the non-synaptic effect that sustains the depolarization of an ictal period of the network (Equation A.6).

$$NS_i^t = a \cdot NSE_i^t - b \cdot NSI_i^t, \tag{A.6}$$

where a and b are parameters that allow for the adjustment of the contributions of the excitation and inhibition on NS_i^t.

In the present model, to characterize the conditions that are favourable to the spontaneous triggering of the ictal period, the neuronal activity is quantified by calculating the average of the probability of each neuron to stay in the "active" state (variable 's' of Equation A.4) during a defined period of time. Based on this quantity, NSE and NSI are calculated:

$$\begin{cases} \dfrac{dNSE_i^t}{dt} = h(t) - q \cdot NSE_i^t \\ \dfrac{dNSI_i^t}{dt} = k(t) - d \cdot NSI_i^t \end{cases}, \tag{A.7}$$

where *h(t)* and *k(t)* are dependent on the state, and *q* and *d* are time constants that govern the recovering rate of NSE and NSI.

To represent the transition between the two states, the state of sustained depolarization was divided into two: the sustained depolarization itself and the state of repolarization. Defining L as the threshold for the action of the non-synaptic mechanisms, the mathematical description of the three states is basically defined by

i) Sustained depolarization state ($s_i^t > L$ and $NSE_i^t > NSI_i^t$):

$$\begin{cases} h(t) = e \cdot \left(s_i^t - 0.25\right) \\ k(t) = f.NSE_i^t \end{cases}, \tag{A.8}$$

with *f* constant and *e* dependent on NSE_i^t, according to

$$e = \bar{e} \cdot (s_i^t - 0.25) \cdot \left(1 - \tanh\left(\frac{NSE_i^t - 18}{0.5}\right)\right) \tag{A.9}$$

where $\bar{e}$ is constant. In this state, *h(t)* represents an increment of Na^+ influx through channels based on the increase of the neuronal discharge. On the other hand, the increment of the intracellular Na^+ increases the Na/K pump activity, thus promoting the inhibitory effect of the electrogenic current, which is represented as an inhibitory effect that is proportional to NSE.

ii) Repolarization state ($NSE_i^t < NSI_i^t$):

$$\begin{cases} h(t) = -g \cdot R^t_{NSE}, & if \quad NSE > 0.1 \\ k(t) = -h \cdot R^t_{NSI}, & if \quad .NSE^t_i > 0.009 \end{cases}, \qquad (A.10)$$

where g and h are constants, and R^t_{NSE} and R^t_{NSI} are functions that contribute to the cell repolarization:

$$\begin{cases} \frac{dR^t_{NSE}}{dt} = l \cdot \left(1 - R^t_{NSE}\right), & if \quad NSE^t_i > 0.1 \\ R^t_{NSE} = 0, & if \quad NSE^t_i \leq 0.1 \\ \frac{dR^t_{NSI}}{dt} = m \cdot \left(1 - R^t_{NSI}\right), & if \quad NSI^t_i > 0.009 \\ R^t_{NSI} = 0, & if \quad NSI^t_i \leq 0.009 \end{cases}, \qquad (A.11)$$

where l and m are constants.

iii) Polarized state ($S_i > L$ with $h(t) = k(t) = 0$).

Synaptogenesis in the network is described considering the following elements: bound synaptic elements, free presynaptic elements, and free postsynaptic elements (Figure 2). New synapse formation is governed by compensation theory[10,42-45], and the changes in the strengths of the synapses follow compensation theory and Hebbian and anti-Hebbian rules[46-48].

The presynaptic elements are represented by *bpr* and *fpr*, and the postsynaptic elements are represented by *bepo* and *fepo* for the bound and free excitatories and by bipo and fipo for the bound and free inhibitories. The sum of the synaptic offers of the *i*th neuron is defined as σ_i, and its variation is $\Delta\sigma_i$. Considering this classification, initially, the sum of the synaptic elements of each neuron *i* is defined as

(i) Sum of the bound excitatory postsynaptic elements: $\sigma_i^{bepo} = \sum_{j=1}^{NE} c_{i,j}$

(ii) Sum of the bound inhibitory post-synaptic elements: $\sigma_i^{bipo} = \sum_{j=NE+1}^{N} c_{i,j}$

(iii) Sum of the free excitatory presynaptic elements $\sigma_i^{fepo} = 0$

(iv) Sum of the free inhibitory presynaptic elements: $\sigma_i^{fipo} = 0$

(v) Sum of the bound presynaptic elements: $\sigma_i^{bpr} = \sum_{i=1}^{N} c_{i,j}$

(vi) Sum of the free presynaptic elements: $\sigma_i^{fpr} = 0$

The formation of a new synapse is dependent on the availability of free presynaptic and free postsynaptic elements (Figure 2). The availability of free presynaptic elements increases when the activity of the corresponding neuron increases, which means that the increased activity of the neuron makes it effective in the network and available for new connections. Therefore, when the activity of the neuron decreases, its free presynaptic elements must be reduced.

The availability of free postsynaptic elements is dependent on their effects. The free excitatory postsynaptic elements must be reduced when the neuron is in high activity and increases when the neuron is in low activity. The inverse occurs for the free inhibitory postsynaptic elements. Therefore, the formation of these elements is guided by an activity regulation to avoid low and high activity levels. Once free presynaptic elements and postsynaptic elements are available, the formation of new synapses is possible and is represented in the model by an increment in the strength of the synaptic interaction between the neurons involved. The strength to synaptic interaction between each pair of neurons of the network is represented by the term connectivity (‘c’). The connectivity of all pairs of the network defines the connectivity matrix C (equation A.1). This synaptogenesis process,

according to the theory of compensation, was implemented computationally according to the morphogenetic rules presented in Table A.1.

In the mathematical model that describes the morphogenetic rules (Table A.1), the parameters $k_{...}^{H}$ and $k_{...}^{L}$ represent the direction of the changes and the characteristic of the morphogenetic rule. Therefore, the degeneration of the free elements is faster than the degeneration of the bound elements:

$$k_{bpr}^{L} = k_{fpr}^{H} * \left(\frac{1.0}{30.0}\right), \tag{A.12}$$

$$k_{bpr}^{L} = \frac{k_{fpr}^{L}}{6} \tag{A.13}$$

To maintain oscillations of the network during morphogenesis, it was assumed that

$$k_{fepo}^{L} = 9 * k_{fipo}^{H}, \tag{A.14}$$

The kinetic constant for the free elements in the high state was given by

$$k_{fpr}^{H} = k_{fepo}^{L} + k_{fipo}^{H}, \tag{A.15}$$

Considering a non-tendentious decrease of synaptic elements,

$$k_{fepo}^{H} = k_{fipo}^{L} = k_{fpr}^{L}, \tag{A.16}$$

$$k_{bepo}^{H} = k_{bipo}^{L} = k_{bpr}^{L}, \tag{A.17}$$

The relationships between the kinetic parameters, described above, allow explaining all of the parameters in a function of only one parameter. Choosing the parameter k_{fipo}^{H} as the adjustment parameter, it was defined that

$$k_{fipo}^{H} = \nu, \tag{A.18}$$

where ν ($0 \leq \nu \leq 0.1$) is a constant that allows controlling the speed of the morphogenetic changes and therefore defines the time constant of these changes.

The decay of presynaptic elements is assumed to be proportional to the strength of the existing connections:

$$c_{i,j} = c_{i,j} + \left(\Delta\sigma_j^{bpr} \frac{c_{i,j}}{\sigma_j^{bpr}} \right), \text{ for all } C_{i,j} \in \mathrm{C}. \tag{A.19}$$

The loss of the postsynaptic elements is computed for the inhibitory and excitatory synapses individually, according to the following equation:

$$c_{i,j} = c_{i,j} + \left\{ \begin{matrix} \Delta\sigma_j^{bepo} \dfrac{c_{i,j}}{\sigma_j^{bepo}}, & j = 1,\ldots,NE \\ \Delta\sigma_j^{bipo} \dfrac{c_{i,j}}{\sigma_j^{bipo}}, & j = NE+1,\ldots,N \end{matrix} \right\}, \tag{A.20}$$

for all $c_{i,j} \in \mathrm{C}$.

The number of pre-synaptic elements previously bound (equal to the number of degraded postsynaptic elements $\left|\delta_{i,j}^{post}\right|$) is transferred to the number of free presynaptic elements.

$$\sigma_j^{fpr} = \sigma_j^{fpr} + \Delta\sigma_j^{fpr} + \sum_{i=1}^{N}\left|\delta_{i,j}^{post}\right| \quad \text{(A.21)}$$

$$\sigma_i^{fepo} = \sigma_i^{fepo} + \Delta\sigma_i^{fepo} \quad \text{(A.22)}$$

$$\sigma_i^{fipo} = \sigma_i^{fipo} + \Delta\sigma_i^{fipo} \quad \text{(A.23)}$$

The recombination is given by

$$c_{i,j} = c_{i,j} + \left\{ \begin{array}{ll} \dfrac{\sigma_j^{fpr}\sigma_i^{fepo}}{\max\left(\sum_{i=1}^{N}\sigma_i^{fepo}, \sum_{i=1}^{NE}\sigma_i^{fpr}\right)}, & j = 1,\ldots,NE \\ \dfrac{\sigma_j^{fpr}\sigma_i^{fipo}}{\max\left(\sum_{i=1}^{N}\sigma_i^{fipo}, \sum_{j=NE+1}^{N}\sigma_j^{fpr}\right)}, & j = NE+1,\ldots,N \end{array} \right\} \geq 0, \quad \text{(A.24)}$$

for all $c_{i,j} \in$ C, where i = 1, ..., N.

Synaptogenesis, according to the Hebb rule, depends on the activities of the cells that are in contact and occurs by one cell contributing to the firing of the other. Thus, the change in the weight of the connection between two neurons i and j was determined by

$$\begin{cases} \Delta c_{ij}^t = \varepsilon\Delta s_i \Delta s_j \eta_{ij}, if\ s_i^t > 0.25\ and\ s_i^t > 0.25 \\ \Delta c_{ij}^t = 0, otherwise \end{cases} \quad \text{(A.25)}$$

where η_{ij} is the number of times the neuron j triggers, an interaction before neuron i, during a morphogenetic step Δt. Considering a maximum limit for the formation of new synapses and for the intensification of the existing synapses, ε depends on the synaptic weight C_{ij}^{t}:

$$\varepsilon_{ij}^{t} = \rho/(1+e^{\left(c_{ij}^{t}-0.4\right)/0.05}) \tag{A.26}$$

where ρ is a constant of proportionality.

The connectivity change, according to anti-Hebb plasticity, was calculated as follows:

$$\begin{cases} \Delta c_{ij}^{t} = -\mu \Delta s_i \Delta s_j \lambda_{ij}, if\, s_i^t > 0.25 and\, s_j^t > 0.25 \\ \Delta c_{ij}^{t} = 0, otherwise \end{cases} \tag{A.27}$$

where μ is a constant, and η_{ij} is the number of times that the postsynaptic neuron is fired before the presynaptic neuron.

The constants of the model are shown in Table A.2.

Table A.1: Morphogenetic rules

Neuron in a high state of activity	Neuron in a low state of activity
$\Delta\sigma_j^{bpr} = 0$	$\Delta\sigma_j^{bpr} = -k_{bpr}^L \lvert\Delta s_i\rvert < 0$
$\Delta\sigma_i^{bepo} = -k_{bepo}^H \lvert\Delta s_i\rvert < 0$	$\Delta\sigma_i^{bepo} = 0$
$\Delta\sigma_i^{bipo} = 0$	$\Delta\sigma_i^{bipo} = -k_{bipo}^L \lvert\Delta s_i\rvert < 0$
$\Delta\sigma_j^{fpr} = k_{fpr}^H \lvert\Delta s_i\rvert > 0$	$\Delta\sigma_j^{fpr} = -k_{fpr}^L \sigma_i^{fpr} \lvert\Delta s_i\rvert \le 0$
$\Delta\sigma_i^{fepo} = -k_{fepo}^H \sigma_i^{fepo} \lvert\Delta s_i\rvert \le 0$	$\Delta\sigma_i^{fepo} = k_{fepo}^L \lvert\Delta s_i\rvert > 0$
$\Delta\sigma_i^{fipo} = k_{fipo}^H \lvert\Delta s_i\rvert > 0$	$\Delta\sigma_i^{fipo} = -k_{fipo}^L \sigma_i^{fipo} \lvert\Delta s_i\rvert \le 0$

Table A.2 – Model parameters (extracted from França et al., 2013)

Equation	Parameter	Value
A.3	α	0 (no stimulation) or 1 (representing stimulation)
	θ	1.0
	β	2.0
A.5	τ	0.19
	κ_1	4.92
	κ_2	2.37
A.6	a	0.8
	b	0.2
A.7	q	2.8×10^{-6} min^{-1}
	d	5.6×10^{-5} min^{-1}
A.8	f	2.8×10^{-3} min^{-1}
A.9	$\bar{e}$	1.1 min^{-1}
A.10	g	2.8×10^{-2} min^{-1}
	h	2.8×10^{-2} min^{-1}
A.11	l	2.8×10^{-6} min^{-1}
	m	1.1×10^{-2} min^{-1}
1	$\bar{\varphi}$	8.0
